# Protocol for behavioral and neural recording during stimulation of the macaque monkey nucleus basalis

**DOI:** 10.1016/j.xpro.2022.101136

**Published:** 2022-01-22

**Authors:** Xue-Lian Qi, Kendyl R. Pennington, Christopher Banerjee, Fernando L. Vale, Sarah K. Bick, Dario J. Englot, Robert S. Turner, Christos Constantinidis, David T. Blake

**Affiliations:** 1 Department of Neurobiology and Anatomy, Wake Forest School of Medicine, Winston-Salem, NC 27157, USA; 2 Department of Neuroscience and Regenerative Medicine, Medical College of Georgia, Augusta University, Augusta, GA 30912, USA; 3 Department of Neurosurgery, Medical College of Georgia, Augusta University, Augusta, GA 30912, USA; 4 Department of Neurological Surgery, Vanderbilt University, Nashville, TN 37212, USA; 5 Department Neurobiology, University of Pittsburgh School of Medicine, Pittsburgh, PA 15213, USA; 6 Department Biomedical Engineering, Vanderbilt University School of Medicine, Nashville, TN 37235, USA

**Keywords:** Model Organisms, Neuroscience, Cognitive Neuroscience, Behavior

## Abstract

We present an experimental protocol to record neuronal activity during intermittent stimulation of nucleus basalis (NB), as macaque monkeys perform cognitive tasks. This protocol includes implantation of electrodes and generator devices to deliver electrical stimulation to NB using multiple approaches in monkeys. Direct stimulation of NB avoids peripheral cholinergic side effects, optimizes timing, and activates non-cholinergic projection neurons. We describe electrode preparation, surgery, and implantation for direct evaluation of how stimulation affects monkeys’ behavior and neuronal activity.

For complete details on the use and execution of this profile, please refer to Qi et al. (2021).

## Highlights

• A protocol for stimulation of the monkey nucleus basalis of Meynert
• Stimulation may be performed in conjunction with neurophysiological recordings
• Alternatively, behavioral data may be acquired during stand-alone stimulation

## Graphical abstract

**Figure:**
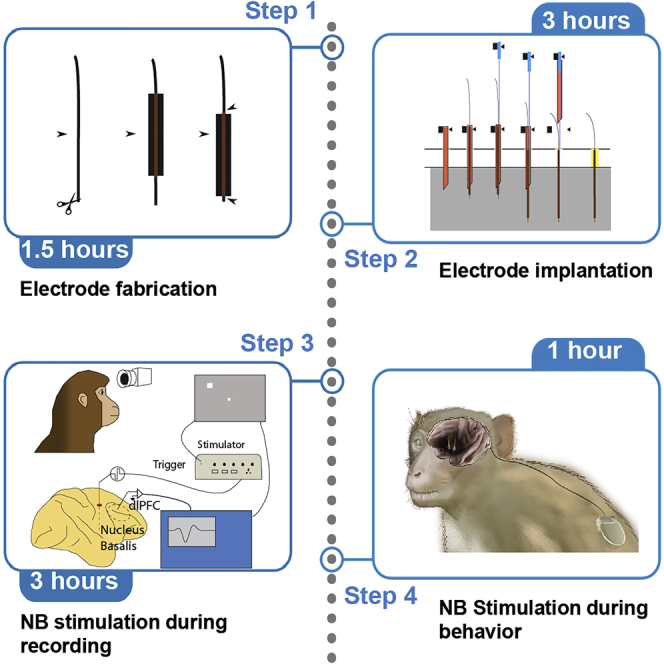

## Teaser

We present an experimental protocol to record neuronal activity during intermittent stimulation of nucleus basalis (NB), as macaque monkeys perform cognitive tasks. This protocol includes implantation of electrodes and generator devices to deliver electronic stimulation to NB using multiple approaches in monkeys. Direct stimulation of NB avoids peripheral cholinergic stimulation, optimizes timing, and activates non-cholinergic projection neurons. We describe electrode preparation, surgery, and implantation for direct evaluation of how stimulation affects monkeys’ behavior and neuronal activity.

## Before you begin

The methods that follow describe methods of implanting Deep Brain Stimulation (DBS) electrodes targeting the Nucleus Basalis (NB) of Meynert in macaque monkeys and recording behavior and/or neural activity during NB intermittent stimulation. Procedures described below followed guidelines by the U.S. Public Health Service Policy on Humane Care and Use of Laboratory Animals and the National Research Council’s Guide for the Care and Use of Laboratory Animals and were reviewed and approved by the Wake Forest University Institutional Animal Care and Use Committee (IACUC), the Vanderbilt University IACUC and the Augusta University IACUC. Animals were maintained in the AAALAC accredited facilities managed by the Animal Resources Program of the Wake Forest University School of Medicine; in the Vanderbilt University facilities maintained by the Division of Animal Care; and the Augusta University animal facilities. Animals were group- or pair-housed in cages whenever compatible peers were available, in facilities housing several individuals of the same species. Methods for behavioral and neural acquisition (which require specialized apparatus, including a microelectrode drive, recording system, and dedicated software) are not described here explicitly; we refer to published studies for more detail. The protocol that follows presents the methods that are necessary to deliver DBS targeting the NB in order to make behavioral and neural data acquisition possible.

## Key resources table

**Table:** 

REAGENT or RESOURCE	SOURCE	IDENTIFIER
**Chemicals, peptides, and recombinant proteins**		

Ketamine	Vedco	383005-08
Dexmedetomidine	Zoetis	122692-5
Isoflurane	Patterson Veterinary	ANADA 200-129
Lidocaine	Taligent	NDC52565-008-14
Atropine	Patterson Vet	07-869-6061
Optixcare/Ophthalmic gel	CLC Medica	12324
Krazy Glue cyanoacrylate	Krazyglue.com	N/A
Quick setting silicone	WPI, Sarasota, FL	Quik-sil
75 μm Pt/Ir, Teflon-insulated wire	A-M systems, Seattle, WA	N/A
Tubing hypodermic	Small Parts, Logansport, IN	29G and 26G
Betadine	Patterson Veterinary	07-836-3379
Nolvasan surgical scrub	Patterson Veterinary	07-803-7207
Tunneling kit	Boston Scientific, Marlborough, MA	tunneler, pigtail wire
Hydrogen Peroxide 3%	Patterson Veterinary	07-801-3480
Sterile Saline Solution (0.9)	Patterson Veterinary	07-800-9721
Dermabond	JNJ Medical	N/A

**Experimental models: Organisms/strains**		

Rhesus macaques (*Macaca mulatta*), 10-14 year old males	Alpha Genesis	N/A

**Software and algorithms**		

MATLAB	MathWorks	R2016-2021a https://www.mathworks.com

**Other**		

APM recording system	FHC, https://www.fh-co.com/	N/A
Headstage preamplifier	FHC, https://www.fh-co.com/	N/A
Stereotax for monkey	Kopf	Model 1430
Microdrive	Alpha-omega	EPS system
Electrode holder/ recording tower	Alpha-omega	Flex MT system
Head-fixation apparatus	Crist Instrument	6-FHP-K1 (SET)
Isolated Pulse Stimulator	A-M system	Model2100
Isoflurane anesthesia system	Engler Engineering	N/A
Heat Pump with heat pad	Gaymar	Model# TP650
Temperature controller, homeothermic blanket system	Augustine Medical	Model# 505
Surgical tools (scalpel, forceps, scissors)	World Precision Instruments	500236
		504473
		191290
Eye scan system	I-Scan	ETL-200
Dental Drill with pedal	Foredom	K.8317
Reward system (pump, water container, drink tube)	Crist Instrument	5-RLD-D1
Impedance meter/multimeter	Fluke Corporation, Everett Washington, fluke.com	Fluke 87V MAX
Resistance spot welder	SunStone, Payson, UT	Sunstone 100 ws
Implantable Pulse Generator	Boston Scientific, Marlborough, MA	Spectra Wavewriter
Capacitor, 10 μF	Digi-Key Electronics	199D106X9035D1V1-ND
Tungsten wire	California Fine Wire	0.18 mm
Hollow Bone Screw	Gray-Matter Research, Bozeman, MT	N/A
Polyimide tubing	Vention Medical, Marlborough, MA	28G
Surgical Loupes	Global-Dental	2.5×
Electrical tape	3M	Scotch Super 33+
Dremel Rotary tool	Dremel	3000 Variable Speed
Dremel Cutoff Wheel	Dremel	545 Diamond Wheel

## Materials and equipment

• Materials for all surgeries:
  Ketamine
  Isoflurane anesthesia system
  Endotracheal tube (Aircare)
  Iris scissors
  Coarse scissors
  Scalpel
  Fine forceps
  Coarse forceps
  Dental drill (Foredom)
  Drill bits – 2 mm tip
  Surgical skin disinfectant: nolvasan/alcohol and betadine
  Dental cement (Paladur, Kulzer)
  Cold sterile saline (0.9% NaCl)
  Lidocaine
  Ophthalmic gel.
  Stereotax (Kopf)
  Heat pump with heat pad.
• For Stimulation electrode fabrication and implantation:
  Magnification stereoscope for visual guidance of electrode descent (or surgical loupes).
  Krazy Glue cyanoacrylate
  Quick setting silicone (Quik-sil, WPI, Sarasota, FL)
  Conductors 75 μm Pt/Ir, Teflon-insulated wire (A-M systems, Seattle, WA)
  Tubing 29G and 26G hypodermic (Small Parts, Logansport, IN)
  Polyimide tubing 28G (Vention Medical, Marlborough, MA)
  Stereotaxic arm and manipulators (Kopf)
  19G Precisionglide syringe needle
  Hollow Bone Screw (Gray-Matter Research, Bozeman, MT)
  Tunneling kit: tunneler, pigtail wire (Boston Scientific, Marlborough, MA)
  Resistance spot welder (SunStone, Payson, UT)
  Implantable Pulse Generator [IPG] (Boston Scientific, Marlborough, MA)
  Stylus: 0.18 mm Tungsten wire (California Fine Wire, Grover Beach, CA)
  Electrical tape
  Dermabond
  Impedance meter
  Dremel Rotary Tool and cut-off wheel

## Step-by-step method details

### Creating the stimulation electrodes (see Figure 1 for this stage)

**Timing: 1.5 h (for a batch of electrodes)**

**Figure 1:**
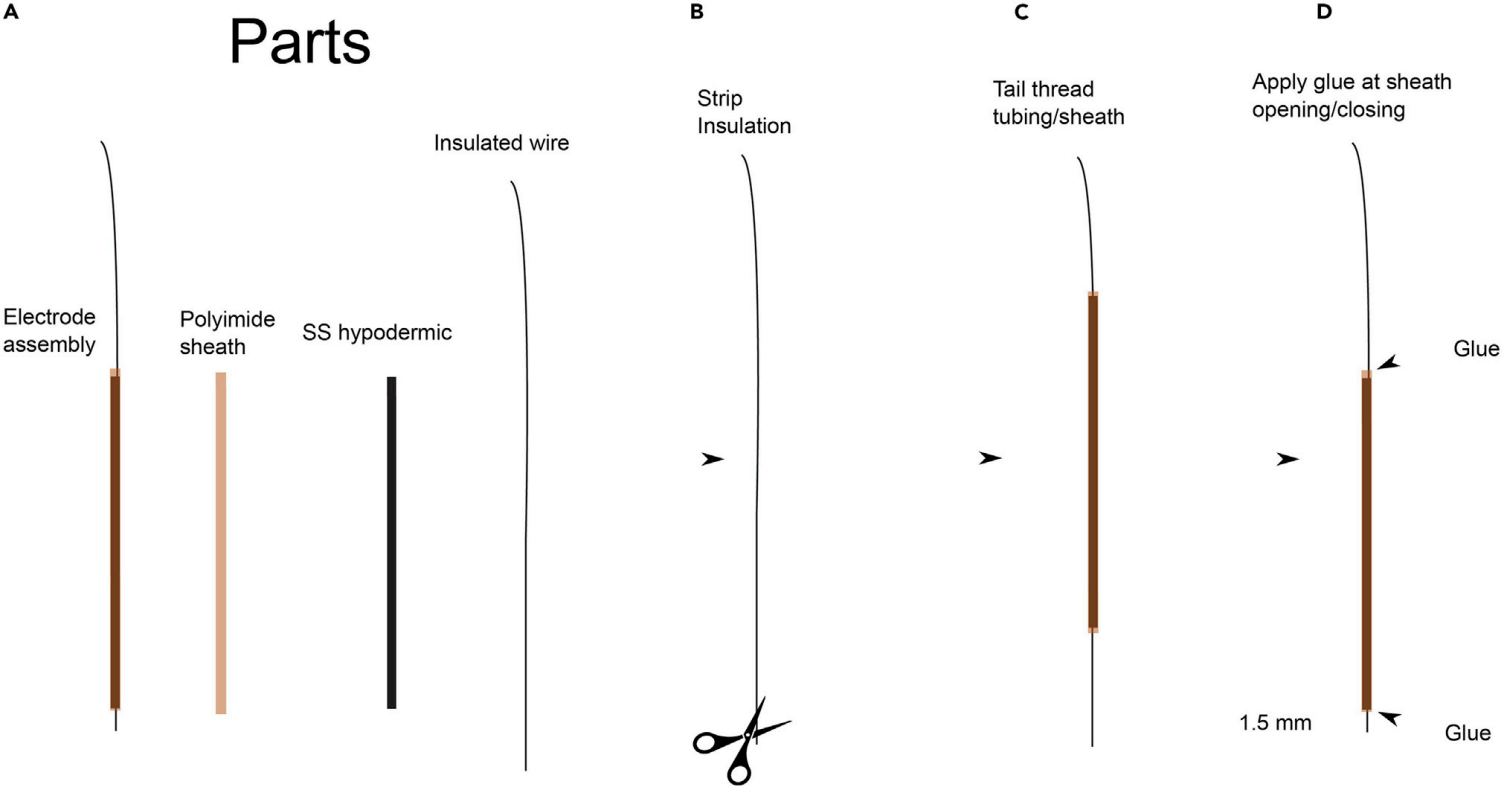
Electrode fabrication (A) Parts include wire, stainless steel hypodermic tubing, polyimide tubing, and cyanoacrylate glue. (B) Strip the insulation from a straight section of electrode for 1 mm. Distance of stripping will determine impedance. (C) Thread the other end of the wire through the tubing and sheath. Thread the tubing into the sheath. Align the tip so it protrudes 1.5 mm from the sheath. (D) Apply cyanoacrylate so it wicks into the sheath on either end.
2. Use iris scissors to carefully remove 1 mm of insulation from the stimulation end to achieve impedances under 1000 Ohms (Figure 1B). For higher impedances, less insulation may be removed.
3. Use a Dremel cut-off wheel to cut and file down the desired amount of hypodermic tubing. Probe the hypodermic tubing with 26G Precisionglide syringe needle to make sure the holes of both ends of the tube have not been blocked by the cutting process. Use file to smooth down both ends of the tubing. Ream the entire section of tubing using tungsten wire.
4. Thread the materials. Locate the stimulation end of the wire (straight) and thread the other end through the metal hypodermic and then the polyimide sheath. The metal hypodermic is threaded through the polyimide sheath (Figure 1C).
5. Pull the wire so less than 1.5 mm protrudes from the polyimide on the stimulating end, with up to 25 mm on the tail end.
6. Glue the wire into the tubing on both ends with cyanoacrylate. The Krazy Glue brand seems to wick into the tubing most effectively. Not all cyanoacrylates are equal in this attribute (Figure 1D).
7. Free insulation from the tail end by mechanical stripping. In some applications, a butane torch may be used, but if the wire is to be welded, mechanical stripping is preferred because it avoids leaving a residue that may interfere with welding.
8. Measure the impedance after removing the insulation. The ideal value is 1000 Ohms at 1 kHz but range of 100–3000 Ω will be acceptable. Typically the reference path electrode is placed in a saline solution, and a 1 kHz signal is pushed from electrode to reference using a current of 1 microamp, and the peak-to-peak voltage measurement is the impedance in megaOhms.
9. Store electrodes in a conventional microelectrode storage box and sterilize them using a low heat method, plasma or gas (McEvoy and Rowan, 2019). The electrode should be steam durable, but the electrode boxes are typically not.
10. The length of insulation removed will dictate the impedance. Preventing irreversible reactions at the metal/brain interface will require charge densities stay within acceptable limits, and delivery of 0.5 mA through a 1 mm length of platinum or iridium with a diameter of 75 μm falls well within these limits (Shannon, 1992). If the impedance measurement is too high, above 10 kOhms, or too low, under 100 Ohms, the tip must be inspected and/or replaced.

***Note:*** These methods have been previously reported briefly (McCairn and Turner, 2009; Turner and DeLong, 2000).

1. Collect materials. A typical electrode uses 31 mm of polyimide tubing, 30 mm of hypodermic tubing, and 55 mm of 75 μm platinum/iridium Teflon insulated wire (Figure 1A).

### Stimulation electrode and implantable pulse generator (IPG) implantation

**Timing: 3 h**

**Figure 2:**
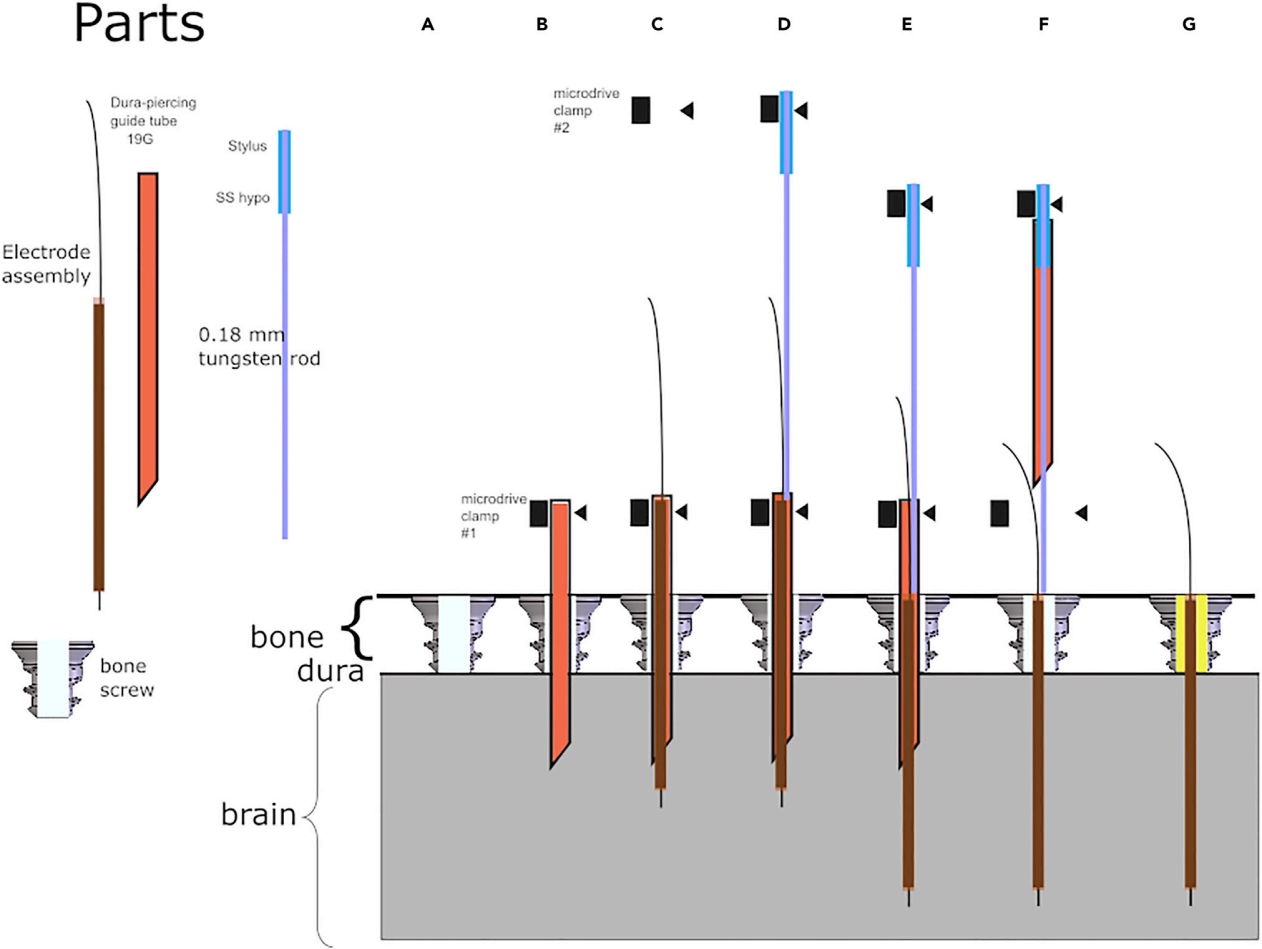
Implanted electrode procedure parts and process (A) A bone screw is installed in the bone allowing a direct path to the target of interest. (B) A guide tube, 23 mm long, is inserted 5 mm into the brain using a stereotaxic positioner, and aligned with the target of interest. (C) The electrode is inserted into the guide tube manually. The length of the electrode, from tip to polyimide tubing tail, allows the tail to be flush with the bone screw when inserted to proper depth. (D) The stylus is held in a second stereotaxic positioner and pushed into the guide tube. The width of the stylus requires the electrode to be advanced as the stylus advances. E. The stylus advances the electrode to depth. F. The guide tube is raised using the first stereotaxic positioner. (G) The stylus and guide tube are removed. The electrode is glued into the bone screw with silicone until there is a hydraulic seal.

**Figure 3:**
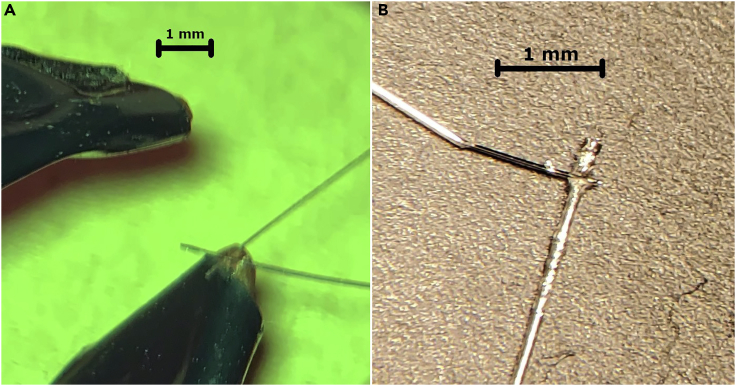
Micro-welding (A) The wires are positioned with uninsulated (stripped) ends of the wire crossed at a 90 degree angle over the tips of the welding tweezers. The tweezers are masked with electrical tape except around the tips. Not shown: the tweezers squeeze the joint between the two wires creating an electrical path from one tweezer face, through both wires, to the other tweezer face. This path creates a thermocouple between the two wires which, with appropriate current pulse, will melt the two wires together. A pulse is delivered. (B) The two wires with a weld are shown. Scale bar = 1 mm. The non-stranded wire used in these images is 0.003” wide at its uninsulated section.

This step describes implantation of stimulation electrodes to deliver stimulation into NB using an IPG (see Figures 2 and 3 for this stage).

11. Before starting surgery, determine target coordinates for implantation of electrode into the NB.
  a. A preoperative MRI is optimal for determining placement. Alternatively, we have used average stereotaxic coordinates when obtaining an MRI was not practical for an animal.
  b. Using a vertically oriented electrode approach, NB is accessible at the following stereotaxic coordinates: 16 mm anterior to the interaural line; 9 mm lateral to the midline; 29 mm below the surface of the cortex.

***Note:*** This position places the electrode 2 mm posterior to the rear margin of the anterior commissure where it crosses the globus pallidus in coronal view.

***Note:*** This landmark is easily viewed on coronal MRI or histological sections and implantation coordinates may be adjusted to target it more accurately in individual cases.

***Note:*** The vertical oriented approach in the macaque monkey minimizes disruption of cortical gray matter and is consistent with most stereotaxic frames and positioners.

12. Prepare the monkey for surgery
  a. Solid food is withheld for 12 h before surgery.
  b. Sedate monkey with intramuscular (i.m.) ketamine (5 mg/kg) and dexmedetomidine (0.2 mg/kg)
    ***Optional:*** the monkey may receive i.m. injections of atropine (0.04–0.06 mg/kg, im) to control secretions.
  c. Shave surgery area comprising the scalp and subscapular area with hair clipper and scrub with Nolvasan.
  d. Intubate and secure endotracheal tube in location.
    **CRITICAL:** make sure the tube is in place by tightening a strip of gauze around the subject’s head.
  e. Put the monkey prone on the surgery table.
    **CRITICAL:** Use a heating pad to maintain the body temperature during surgery, aiming for 36°–38°C.
  f. Anesthetize with isoflurane, 3% for induction, 1.5% for maintenance delivered in 2 L/min oxygen. Monitor anesthesia by means of SpO2, Heart Rate, Respiration Rate, End-title CO_2_, temperature.
  g. Administer ophthalmic ointment in both eyes of the animal to protect the corneas from drying and abrasion.
  h. Secure monkey on stereotax.

13. Disinfect shaved area: clean the surgical site with antiseptic scrub.
14. Administer lidocaine subcutaneously at the surgical site (2 cc of 2% solution).
15. Cut a flap of scalp through the galea to expose the bone.
16. Drill a hole according to coordinates obtained from MRI.
  a. Implant a bone screw with a radially centered hole in it.

**CRITICAL:** The bone screw and hole should both be performed in stereotaxic vertical orientation and not orthogonal to the bone angulation and should be vertically above the desired stimulation location.

***Note:*** Bone screws were custom designed with Gray-Matter Research, and are 4.5 mm long, with a 1.85 mm diameter through hole. The threads are bone screw pitch, 1.25 mm spacing.

***Note:*** The bone screw seats well in the cranial bone and creates a minimal defect in the sealing of the cranium that can be easily sealed. It also enables later removal of the electrode should it become necessary for repositioning or MRI imaging (Figure 2A).

17. Lower a 19 ga. sharp hypodermic guide tube using the manual rotational drive on the stereotaxic micropositioner. The tip should be carefully advanced at least 5 mm below the dura mater. The guide tube is advanced in stereotaxic vertical orientation, and acts as the primary targeting vector for the electrode tip. Check its orientation carefully. Measure the length of the electrode to be inserted, and the length of its wire tail (Figure 2B).

**CRITICAL:** The guide tube for this procedure is 23 mm long, which is important for its later removal. The guide tube needs to be long enough to pass the dura, and short enough that it can be later removed while visualizing the tail end of the electrode.

18. Insert the stimulation electrode manually into the guide tube, and push the electrode until the polyimide sheath is fully within the guide tube. Insert the stylus into the guide tube and advance electrode to appropriate depth using a clamped manipulator with Vernier scale or microdrive. The guide tube is then raised while the stylus depth is maintained (Figures 2C–2F).

***Alternatives:*** Use a longer guide tube in a stylus free preparation. In that scenario, advance the electrode until its shank is flush with the rear of the guide tube. Then, remove the guide tube. Advance the electrode to target depth. This approach will also work if the tail of the electrode shank will be approximately positioned at the top of the bone screw, and will thus provide its own guidance on depth.

19. Check tail length of electrode after dropping to confirm depth (Figure 2G). Glue electrode into bone screw head using one drop of quick curing silicone to stabilize the electrode in the bone screw.
20. Repeat for the second electrode.
21. A surgical site for the IPG is located on the back, caudal enough to avoid touching the scapula, and lateral enough to avoid touching the spine. Make a horizontal incision perpendicular to the spine. Dissect a pocket bluntly in the subcutaneous space to fit the IPG caudal to the incision.
22. Use a tunneling kit to tunnel from the head surgical location to the back incision.
  ***Note:*** Large hemostats (8” or 12”) are useful surgical tools before moving to the tunneling tool.
  a. The tunneling tool is advanced subcutaneously from scalp incision to IPG pocket.
  b. The straw is left in the tunnel, and the pigtail wire inserted through the straw.
    ***Note:*** The pigtail wire is a Boston Scientific product, and contains an industry standard 8 lead connector on one end, and eight stranded wires on the other end.
  c. Once the pigtail is inserted, the straw is removed.

23. Welding. See Figure 3.
  a. Use a Sunstone 100 ws single pulse resistance spot welder, along with its Tweezer hand piece weld attachment.
  b. Set the energy to 4% with a 0.45 ms pulse width.
  c. Use electrical tape to mask the Tweezer except where the two faces come together.
  d. The electrode wire tail, and pigtail wire, both mechanically stripped of insulation, are overlaid at a 90 degree angle in between the tweezer faces so that the only current path is from one tweezer face through the joint between the wires to the other tweezer face.
    ***Note:*** Typically two people are needed to hold the Tweezer and both wires. One person holds both wires at 90 degree angles, while the other positions the Tweezer.
  e. Then, a pulse can be used to spot weld the wires. Wire alignment on the Tweezers is critical, and practice prior to surgery recommended. These welding settings are specific to the wire diameters and composition used in this study.
    **CRITICAL:** The welding step must not be performed while the IPG is connected to avoid damage to the IPG.

24. Check channel number using a multimeter. The connectivity between the weld and the pigtail connector contacts can be used to determine which channel is attached to the electrode. A nonsterile multimeter and two sets of sterilized alligator clip wires, and two pairs of forceps, are used to find the connector contact matching each weld.
25. Check impedance.
  a. Plug the pigtail connector into the IPG, and place IPG into a previously created subcutaneous pocket.
  b. Test impedance using a Boston Scientific tablet, which is a specific accessory associated with their IPGs.

***Note:*** Impedances in the 4 kOhm range or lower are expected. Impedances above 10 kOhm indicate replacement of the electrode should occur.

***Note:*** Often the bone screw will need to be removed to facilitate removal, if needed. Once both electrodes are welded and verified, move on.

26. Place a drop of silicone on the skull for each of the two welds. Hold the uninsulated section of the welds in the silicone to insulate them.
27. Flush head and IPG surgical sites with 3% hydrogen peroxide diluted 50:50 in 0.9% NaCl irrigation solution. Insert IPG into the subcutaneous pocket. Coil extra pigtail wire under IPG. Silk sutures may be used to secure the IPG. Subcutaneous silk sutures should be used to secure the pigtail at the cranial location to avoid pigtail creep.
28. Suture both sites closed using interrupted 3-0 Vicryl for the galea (scalp) or cutaneous (back) layer, and a subcuticular running 4-0 Monocryl for skin, and apply Dermabond to the incisions.

**Figure 4:**
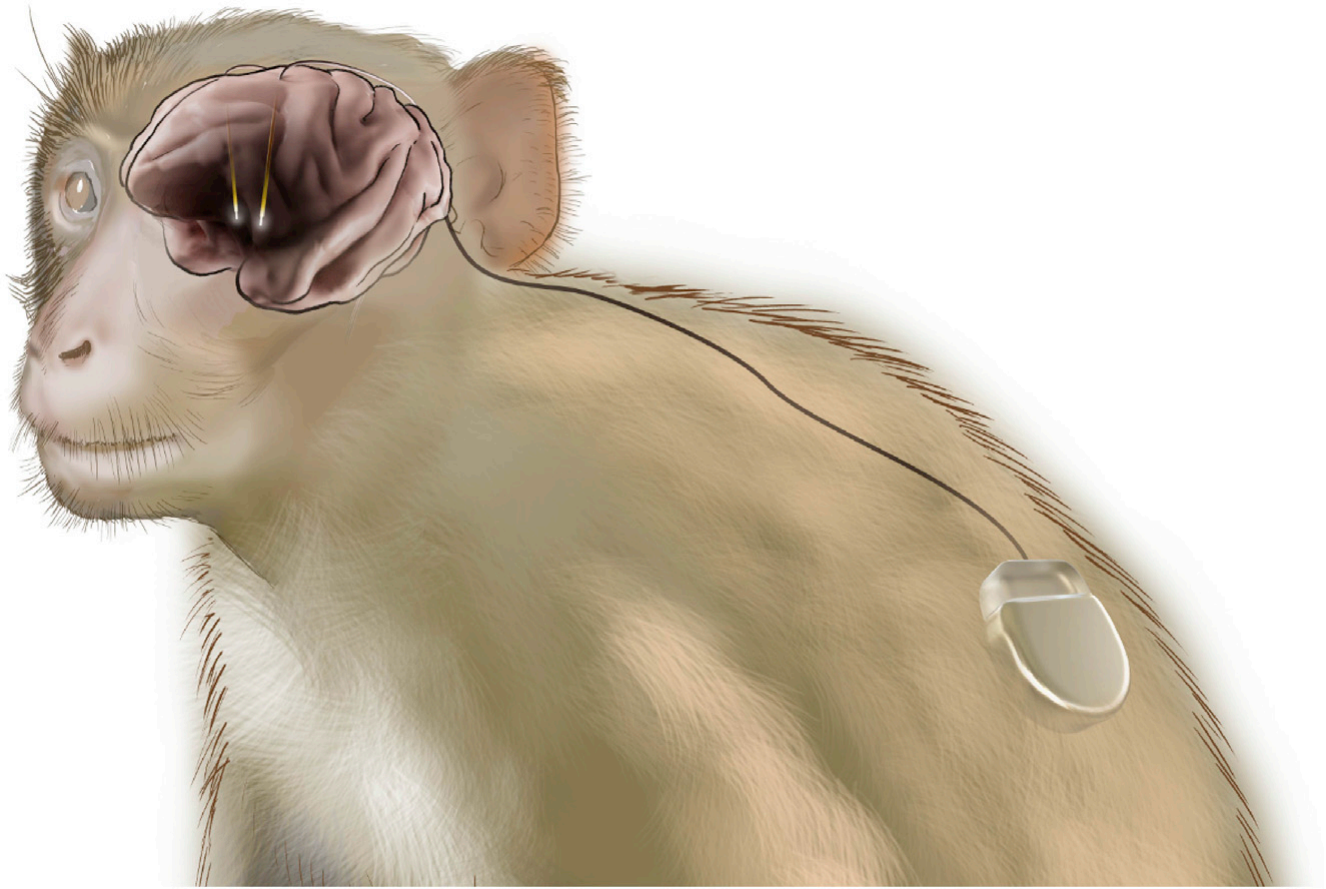
Schematic figure of implanted rhesus monkey Locations of implanted electrodes, route of pigtail wire from cranial surface to subscapular subcutaneous implantable pulse generator are shown.

The relative positions of pigtail wire, electrodes, and IPG are shown in Figure 4.

29. Treat the monkey post-operatively with opioid analgesics (e.g., buprenorphine) for up to 72 h, followed by nonsteroidal anti-inflammatory drugs (e.g., ketoprofen) for up to 1 week.

***Note:*** Suspected infections around the wound margin may (rarely) require antibiotic treatment. Suspected brain edema may require treatment with steroid drugs (e.g. dexamethasone).

### Stimulation electrode implantation – Stimulation through external connector

**Timing: 3 h**

In some preparations it is desirable to have the connector to the electrode externalized through an acrylic cap that will also be used to anchor recording chambers. This alternate approach describes implantation of stimulation electrodes to deliver stimulation into NB using an external stimulator, connected to the electrodes through a removable connector, in dental cement or acrylic.

Repeat steps 1–21 of electrode fabrication and stimulation electrode implantation, above.

30. Once both electrodes are implanted, and wires are loose, the wires are attached to the connector via soldering. A bare piece of platinum wire is wrapped around a separate bone screw to act as the ground.
31. The wires and connector are encased in a bone-cement head cap, adhering to the screws.
32. Implantation of additional screws may be needed for added stability. Alternatively, this procedure may be performed in monkeys who already have a headpost/recording cylinder implanted and covered by bone-cement. The bone cement that has been added to stabilize the electrodes then attaches to the rest of the implant.
33. A cap may be added to protect the connector; all parts of the electrode are encased in acrylic at the end of the procedure.

### Combined stimulation – Neural recording

**Timing: 2 h**

**Figure 5:**
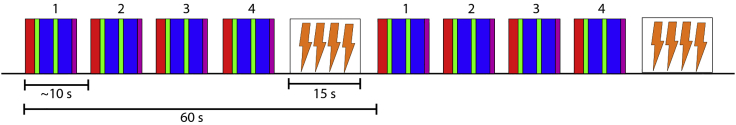
Trial structure for combined behavioral and neural recording Each minute consists of 45 s of behavioral trials, which may contain different events such as fixation periods, visual stimulus presentations, delay periods, and response periods, represented by different color rectangles. The number of trials that take place in each period depends on the duration of each trial (4 trials are shown in this example). Behavioral trials are interleaved with 15 s periods of stimulation, when no behavior is performed, and animals are conditioned simply to pause. Comparisons can be made between blocks of trials involving stimulation during the 15 s pause period, and blocks of trials not delivering stimulation (sham). No trials are performed during stimulation because voltage recordings are contaminated in that period.

Monkeys that have already had cranial implants that allow neurophysiological recordings prior to the stimulation electrode implantation (e.g., headpost and cranial well that allows insertion and removal of micro-electrodes through a craniotomy into the brain) may undergo simultaneous deep brain stimulation and neural recording with a data acquisition system such as the APM recording system. The first step in this experiment is to load the electrodes into the micro-electrode microdrive and prepare and test the neural recording system (see Figure 5 for this stage).

34. Head fix monkey and put recording stage on and connect to preamplifier
35. Test impedance of stimulation electrode by connecting external connector to an impedance meter. The use of implanted electrodes includes the possibility of failure, and regular impedance checks are necessary to ensure connectivity (see also problem 1 in Troubleshooting section).
36. Connect stimulator to stimulation electrode (e.g., isolated pulse stimulator Model 2100, A-M Systems, Sequim WA).

***Note:*** Connect a 10 μF capacitor in series to the stimulating electrode to prevent net charge accumulation.

37. Advance the micro-electrodes into the brain (e.g., prefrontal cortex) through the cranial well and stop when neural signals have been isolated.
38. Start behavioral task, implemented in the MATLAB environment e.g., as described in (Meyer and Constantinidis, 2005) or equivalent. Behavioral program will trigger stimulation.

**CRITICAL:** During the time interval of electrical stimulation, electrical artifacts are visible in the neurophysiological data acquisition. These make recording of neurophysiological signals anywhere in the brain impossible, for a brief period of time (<1 s). It is imperative therefore that the behavioral task and data acquisition be structured so that artifacts do not interfere with recordings during task execution. At the same time, presence of artifacts in the neurophysiological recording when delivering electrical stimulation provides confirmation that the stimulation circuit is closed and the current application is effective.

39. The recommendation is to construct a recording task consisting of 45 s of trials, with 15 s pauses interleaved. During the 15 s pauses, biphasic stimulation can be delivered to one or both hemispheres at intensities up to 0.5 mA, with 100 microsecond phase length, biphasic charge balanced pulses. Good effects of stimulation have been documented at 80 pulses per second during that 15 s period (Liu et al., 2017; Qi et al., 2021).
40. Retract the recording electrodes first from the recording chamber; when task is done then power off and disconnect the stimulator.
41. Clean cranial well with sterile saline. Remove guide tube and electrodes, clean with alcohol followed by distilled water and sterilize with UV irradiation.

### Combined stimulation/IPG – Behavioral recording (see Figure 6 for this stage)

**Timing: 1 h**

**Figure 6:**
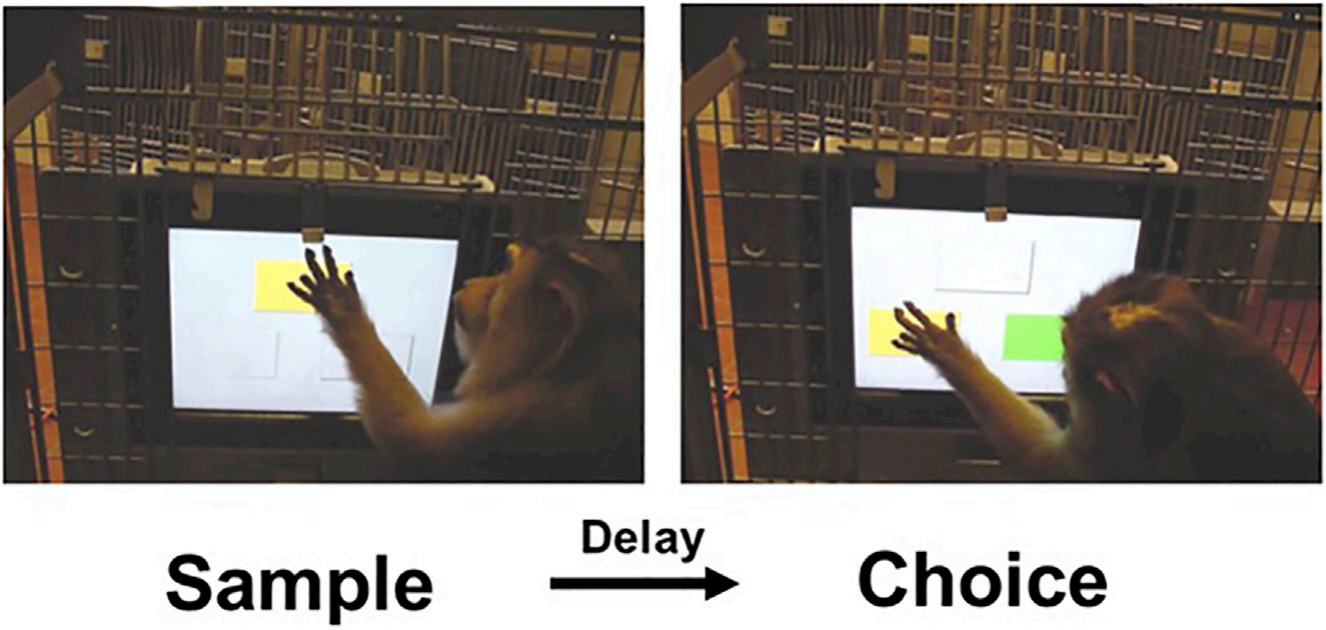
Monkey interacting with the apparatus Monkey performing a delayed match to sample working memory task in its home cage using a cage-mount touchscreen apparatus.

Monkeys that have not had cranial implants that allow neurophysiological recordings, or when obtaining behavioral measures alone is adequate, NB stimulation can be performed around behavioral recording. An example is shown in Figure 6.

42. Test impedance of stimulation electrode. Connections between the implanted electrodes and the IPG may fail, and regular impedance checks are necessary to ensure connectivity. The IPG system allows impedance testing remotely.
43. Create a program of stimulation. Intermittent stimulation comprises biphasic pulses delivered to one or both hemispheres at intensities up to 0.5 mA, with 100 μs phase duration. Positive effects of stimulation have been documented at 80 pulses per second during that 15 s period. The activating function is proportional to the square root of the current (McIntyre et al., 2004; Murasugi et al., 1993; Stoney et al., 1968), and currents of 200–500 microamps per pulse have been used in the macaque
44. Prepare and test behavioral control system.
45. Apply stimulation for a period of 1 h, starting stimulation at the same time the monkey engages with a behavioral task.
46. Daily sessions of stimulation during the task may alternate with sessions beginning after the 1 h of stimulation.

## Expected outcomes

**Figure 7:**
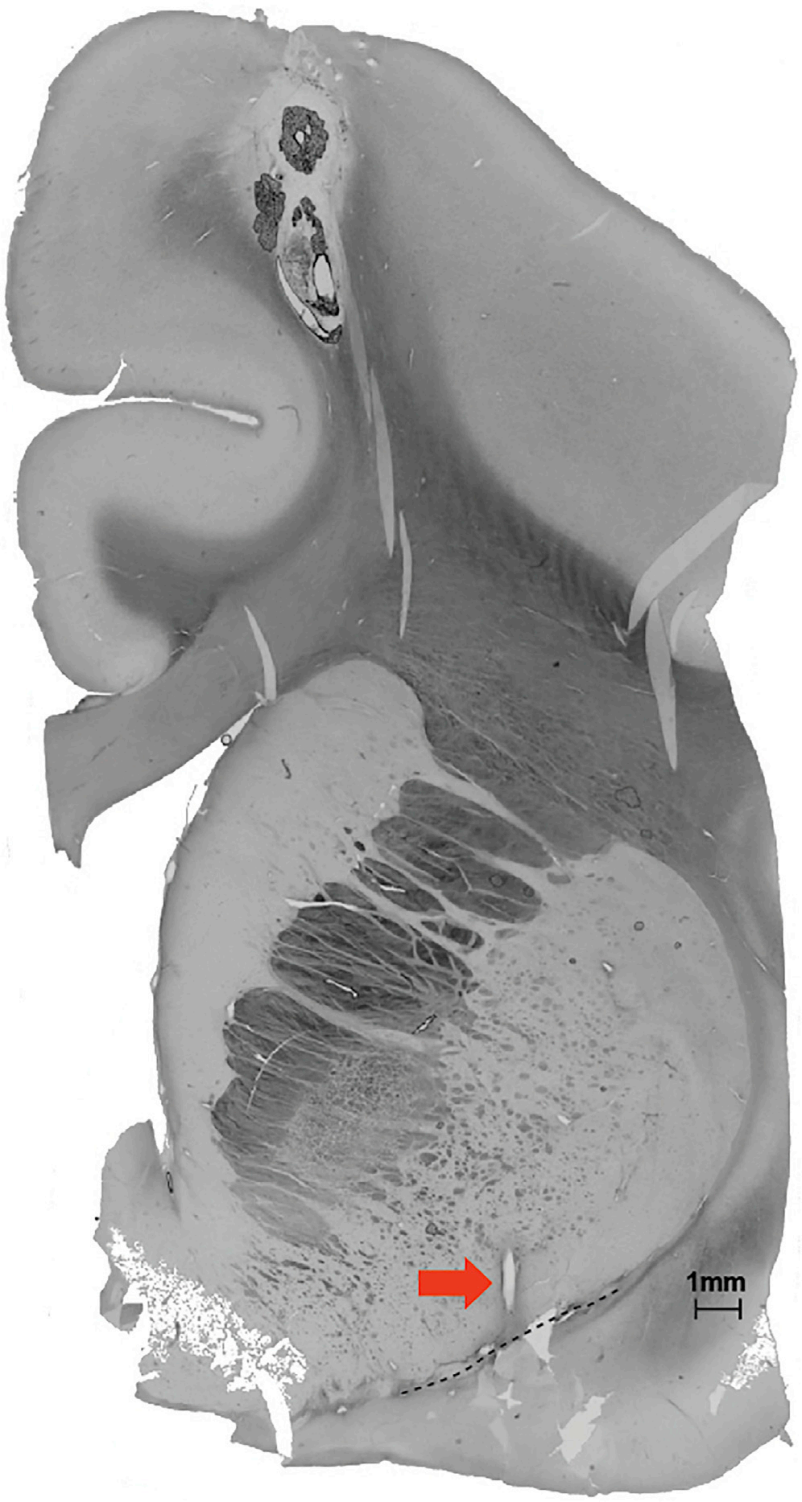
Anatomical confirmation of electrode placement Histological section displaying the most inferior track of the electrode (red arrow). The dashed line marks the floor of the Nucleus Basalis. Scale bar: 1 mm. Adapted from Liu et al. (2017).

Absolute verification of electrode placement requires histological verification that the tip is positioned in the floor of the globus pallidus, with anterior-posterior position determined by where the anterior commissure crosses the globus pallidus (Figure 7). Electrodes may be imaged using CT postoperatively to validate placement, but final placement should be verified histologically. We have demonstrated that these stimulation protocols improve working memory performance (Liu et al., 2017; Qi et al., 2021), and sustained attention performance (Liu et al., 2018), generate long-term neuroplasticity based effects (Liu et al., 2017), and desynchronize the EEG (Qi et al., 2021). During recording, for a successful stimulation we will expect to see artifacts from recording electrodes during each stimulation pulse. Animals show no observable reactions to this form of brain stimulation, and in particular no consistent pupil dilation or contraction occurs.

## Limitations

Practically, regular verification of electrode impedance and visualization of stimulation artifacts when recording are the primary methods used to ensure the circuits are intact. Reliable positioning of the animal into the stereotax using aural and orbital ridge landmarks is critical to success, and can be practiced with plastic model skulls to verify reproducibility. This process is the largest potential source of error in positioning. Enabling the tail end of the electrode to be visible in acrylic cap preparations allows verification of depth, and behavioral impairment via high frequency stimulation (Liu et al., 2017) is robust and useful to improve confidence in electrode positioning. Better biomarkers of appropriate electrode placement prior to histological verification are an area for improvement.

## Troubleshooting

### Problem 1

When the electrode is coupled to an acrylic cap, wiring may break; for this reason impedance checks are performed manually each day to verify an intact circuit (step 35).

### Potential solution

We always manually check the impedance of an actual 10 kOhm resistor before checking the implanted animal to ensure the impedance checking procedure is competent. If the circuit is broken, the most likely point of failure is in the wire where it exits the electrode tubing. We have increased the wire diameter from 50 to 75 μm from the same supplier and this problem has become rarer.

### Problem 2

Stimulation parameters may not be implemented correctly (step 39).

### Potential solution

The stimulation pulses should be placed on an oscilloscope during sessions to verify the voltage is negative at the electrode first (cathodal biphasic pulses).

### Problem 3

It may not be possible to synchronize stimulation perfectly with events of the behavioral task (step 46).

### Potential solution

We have reconstructed behavioral performance relative to time of stimulation onset in multiple experiments in each of our labs, and found performance is remarkably insensitive to the timing of the trial relative to stimulation during intermittent stimulation.

## Resource availability

### Lead contact

Further information and requests for resources and reagents should be directed to and will be fulfilled by the lead contact, David T. Blake, dblake@augusta.edu.

### Materials availability

No new mouse lines or materials were generated for this protocol. Boston Scientific pigtail wires and IPGs were provided by Boston Scientific through an agreement modeled on the BRAIN Initiative Public-Private Partnership templates.

## Data Availability

No new data or code was generated for this protocol.
